# Gender bias of antisocial and borderline personality disorders among psychiatrists

**DOI:** 10.1007/s00737-024-01519-0

**Published:** 2024-10-04

**Authors:** Beren Özel, Ezgi Karakaya, Fazilet Köksal, Ali Ercan Altinoz, Imran Gokcen Yilmaz-Karaman

**Affiliations:** 1 Department of Psychiatry, Faculty of Medicine, Baskent University, Ankara, Türkiye; 2 Department of Psychiatry, Faculty of Medicine, Ankara University, Ankara, Türkiye; 3 Department of Psychiatry, Faculty of Medicine, Eskişehir Osmangazi University, Eskişehir, Türkiye; 4 Department of Life, Health and Environmental Sciences, University of L’Aquila, L’Aquila, Italy

**Keywords:** Gender bias, Sexism, Personality disorder, Antisocial, Borderline, Diagnostic and statistical manual of mental disorders

## Abstract

**Purpose:**

Antisocial Personality Disorder (ASPD) and Borderline Personality Disorder (BPD) challenge mental health professionals with similar maladaptive behaviors. However, these two disorders differ regarding available evidence-based treatments. The Diagnostic and Statistical Manual of Mental Disorders (DSM) has been criticized as being gender-biased diagnostic construct. The present study aimed to determine the gender bias of ASPD and BPD among Turkish psychiatrists.

**Methods:**

Three case vignettes were randomly presented as male or female to the psychiatrists online. The first case was a patient with schizophrenia case to confirm the participant’s ability to diagnose. The second case was a patient with ASPD, and the third one was a patient with BPD.

**Results:**

Two hundred fifty participants diagnosed the first case correctly (*n* = 250). The results with statistical significance demonstrated that a female case with ASPD was 5.1 times more likely to get misdiagnosed than a male case with ASPD (p˂0.0001).

**Conclusions:**

Categorical classification of personality disorders in DSM leads to gender bias in in the diagnosis of ASPD and BPD. The present study shows that female cases with ASPD are misdiagnosed as BPD which may result in treatment attempts without evidence.

**Supplementary Information:**

The online version contains supplementary material available at 10.1007/s00737-024-01519-0.

## Introduction

The reference book The Diagnostic and Statistical Manual of Mental Disorders (DSM) on mental health and brain-related conditions and disorders has provided many conveniences, from clinical practice to insurance systems, but it is also known with some biases. Bias occurs when diagnostic criteria or clinical judgments are more valid for one group than another (Garb 2021). The personality disorders section of the DSM has faced criticism from scholars and clinicians over the past few decades due to perceived gender bias and gender-related differences (Samuel and Widiger 2009). Starting with Kaplan’s paper in 1983 (Kaplan 1983), numerous subsequent studies have mentioned concerns regarding gender bias in the assessment and diagnosis of personality disorders (Samuel and Widiger 2009). When making a diagnosis antisocial personality disorder (ASPD) and borderline personality disorder (BPD) seem to be the most challenging personality disorders with their similar maladaptive behaviors (Chun et al. 2017). Clinicians may diagnose women with BPD, while men are diagnosed with ASPD, when they apply with similar problems. (Skodol et al.,2003)

**ASPD** is a condition characterized by disregarding and violating the rights of others (American Psychiatric Association [APA] 2022). In clinical settings, ASPD is prevalent 3–30%, and more common in males than in females (Schulte Holthausen and Habel 2018). ASPD may also be present as a comorbid diagnosis when other complaints are prominent (APA 2022; Paris et al. 2013). While comorbid substance use disorders are more common in men (APA 2022), women with ASPD were found to be more likely to have negative experiences of childhood, sexual abuse and comorbid mood disorders (Alegria et al. 2013; Sher et al. 2015). **BPD**, a predominately “female” personality disorder with a sex ratio of 3:1, is a typical pattern of marked impulsivity and inconsistency in interpersonal relationships, self-perception and affect, beginning in early adulthood and presenting in various contexts (APA 2022). The prevalence of BPD is approximately 6% in primary care, 10% in individuals seen in outpatient mental health clinics, and approximately 20% in psychiatric inpatients (APA 2022). Since the introduction of personality disorders in DSM in the 1980s, BPD has often been perceived as a female-predominant disorder in both research and clinical contexts. However, more recent research suggests that prevalence rates may not be significantly different between males and females (Qian et al. 2022; Sansone and Sansone 2011). The clinical features of men and women with BPD appear similar; potentially higher degrees of externalizing behaviors in males and internalizing behaviors in females (APA 2022; Qian et al. 2022) which may be related to traditional gender roles. Gender roles are the stereotypical emotions, cognitions, and behaviors associated with being male or female and are presumably acquired through socialization. Research has shown that traditional gender roles (masculinity/instrumentality vs. femininity/expressivity) mediate a substantial portion of sex differences in both externalizing and internalizing problem behaviors (Kulis et al. 2010).

## The impact of diagnostic biases on treatment approaches for ASPD and BPD

In 1988, Lewis and Appleby showed that psychiatrists have negative attitudes toward patients diagnosed with personality disorders (Lewis and Appleby 1988). The diagnostic label significantly influences psychiatrists’ attitudes more than the patient’s gender or class. Indeed, diagnoses of personality disorders pose barriers to stigmatization and effective care among healthcare providers (Chartonas et al. 2017).

Although ASPD and BPD differ largely in prevalence among males and females, clinician bias can easily affect the diagnosis due to the overlap of symptoms for both disorders. The diagnostic process is susceptible to bias, potentially influenced by social and gender stereotypes, such as perceiving women as more emotional and neurotic and men as more assertive (Schulte Holthausen and Habel 2018). Sprock suggests that if a diagnostic criterion is deemed more pathological in women than in men, clinicians may be inclined to apply that criterion more readily to women (Sprock et al. 1990). That introduces the concept of assessment bias, where the diagnostician’s interpretation of the item or criterion is influenced by preconceived notions (Morey et al. 2002). Moreover, diagnostic constructs themselves can perpetuate gender bias. Morey et al. (2002) argue that biases emerge not only in the diagnostic process but also in the very constructs used, reflecting sexist characterizations of gender. The gender composition of DSM working groups adds another layer to this issue, with concerns raised about whether diagnostic criteria might be inadvertently shaped by a predominantly masculine perspective within DSM working groups (Disney 2013). Determining the factors contributing to misdiagnoses, it becomes evident that both the criteria embedded in the DSM and potential internalized gender bias among clinicians contribute significantly to the challenge of diagnostic biases.

However, accurate differentiation between ASPD and BPD is essential to optimize treatment approaches, ensuring a focus on individual needs while minimizing the risk of gender bias. A recent Cochrane systematic review showed limited research on treatment programs targeting antisocial behavior, and no evidence-based treatment is available for ASPD (Gibbon et al. 2020). Although symptom remission of ASPD may occur in middle adulthood, the prognosis of treatment is poor. Therefore, treating individuals with ASPD must be tailored to each patient to promote change (Martens 2000). Furthermore, a diagnosis of ASPD is often an exclusion criterion for admission to mental health services. Patients with ASPD may be unable to access treatment even if they seek help for comorbid psychiatric disorders (van Dam et al. 2022). The prognosis of BPD treatment appears to be better. Different treatment modalities can treat BPD, including Dialectical Behavioral Therapy (DBT), Mentalization-Based Therapy, Transference-Focused Psychotherapy, and Schema-Focused Therapy. In particular, the findings suggest that the effects of DBT in treating BPD are maintained for at least 1 to 2 years postintervention (Gillespie et al. 2022). On the other hand, psychotropic drugs have little effect on the underlying aspects of both disorders and are used for symptoms such as impulsivity (Kantojärvi et al. 2020; Timäus et al. 2019).

Individuals with ASPD and BPD differ in clinical representation regarding treatment interventions, safety measures, and treatment effects. Therefore, determining whether bias among psychiatrists influences the diagnosis of these disorders is essential to ensure that patients receive appropriate mental health treatment.

The present study aims to determine the extent to which gender-based cognitive biases are present when diagnosing antisocial personality disorder and borderline personality disorder among Turkish psychiatrists and to raise awareness in mental health professionals with the research results. We hypothesize that, among Turkish psychiatrists, a female case presenting with symptoms indicative of ASPD will be more likely to face misdiagnosis compared to a male case with ASPD. Simultaneously, we posit that a male case exhibiting symptoms aligned with BPD will be more likely to experience misdiagnosis compared to a female case with BPD.

## Method

The present study is a cross-sectional study, testing if psychiatrists’ clinical diagnoses regarding borderline and antisocial personality disorders contain gender bias using case vignettes. The first case vignette aimed to test the participant’s diagnostic competency. The following two case vignettes aimed to provide information on gender bias in diagnosis. The gender of all cases was randomly assigned as female or male.

### Sample

The present study utilized a convenience sampling method. The study sample consists of consultant psychiatrists and psychiatry residents. The inclusion criteria were volunteering to participate and being an adult psychiatrist or a psychiatry resident with the experience of at least one year of psychiatry residency. Exclusion criteria were unable to give the correct answer for the control case.

The research link was shared in Turkish-speaking psychiatrists’ social media groups.

## Procedure

Approval of the study was obtained from the Eskişehir Osmangazi University Social and Human Sciences Human Research Ethics Committee with decision number 2023-04/10. The informed consent and data collection forms were transformed into an online survey via the program Qualtrics. The reason for choosing this program was its allowance for random allocation.

The study was introduced in the e-mail group of psychiatrists in Turkiye and the link to participate in the study was shared in the group. In addition, the survey link was disseminated via Turkish-speaking psychiatrists’ social media accounts such as Instagram, Twitter, and WhatsApp.

Those who gave consent to participate in the study filled out the measurement tools and provided diagnostic information for the three vignettes they encountered. Each participant read and diagnosed three vignettes. While the vignettes remained same for each participant, gender of the vignettes were randomly assigned by the system.It took 5–8 min to complete the survey.

## Measures

### Sociodemographic information form

This form was prepared by the researchers to collect information about the participants’ age, gender, occupational status, and professional experience.

### Vignettes

The study presented three vignettes from the book DSM-IV Case Studies (Frances and Ross 1996). The vignettes were first translated into Turkish and shortened, then sent to ten experienced psychiatrists for their feedback. In line with the feedback, the vignettes took their final form. The final version of the vignettes is available in the additional documents section. While the first vignette aimed to test participants’ diagnostic competencies, the remaining two vignettes were used to measure gender bias in participants’ diagnoses.

The first vignette was a patient with schizophrenia. After reading the vignette, the participant was expected to guess the diagnosis. The options for diagnosis were: (A) Schizophrenia, (B) Bipolar disorder, (C) Anxiety disorder, (D) Depression, (E) Obsessive-compulsive disorder.

The second vignette was a case of antisocial personality disorder.

The third vignette was a case of borderline personality disorder. The same case was randomly presented to the participants as male or female. Participants were asked to diagnose the case, and the diagnosis options for the second and third cases were: (A) Antisocial personality disorder, (B) Borderline personality disorder, (C) Histrionic personality disorder, and (D) Narcissistic personality disorder.

### Statistical analysis

IBM SPSS version 26 was used for data analysis. Descriptive statistics were presented as means and standard deviations for continuous variables and frequencies and percentages for categorical variables. Independent samples t-tests were used for continuous variables in binary group comparisons, and chi-square tests were used for categorical variables. The relationship between gender and accurate personality disorder diagnosis was examined using odds ratios: The clinician’s diagnosis on Vignette 2 (the patient with ASPD) and Vignette 3 (the patient with BPD) was dummy-coded as “correct diagnosis” and “incorrect diagnosis.” Without gender bias, the authors would expect no differences in the percentages of correct diagnoses when the patient’s gender changes but the vignette remains the same. Thus, for Vignette 2 and Vignette 3, at the same time for the cases of ASPD and BPD, binary logistic regression was performed to test if the patient’s gender predicts the correct diagnosis. Binary logistic regression analyses were also utilized to evaluate predictors of misdiagnoses.

Post-hoc power calculation was performed. Sample contained 250 participants, and calculated Odds ratio was 5.1. Alpha level was assumed as 0.05. Finally Cohen’s h value was calculated as 1.04; that means an adequate effect size.

A statistically significant p-value was considered to be 0.05.

## Results

**Fig. 1:**
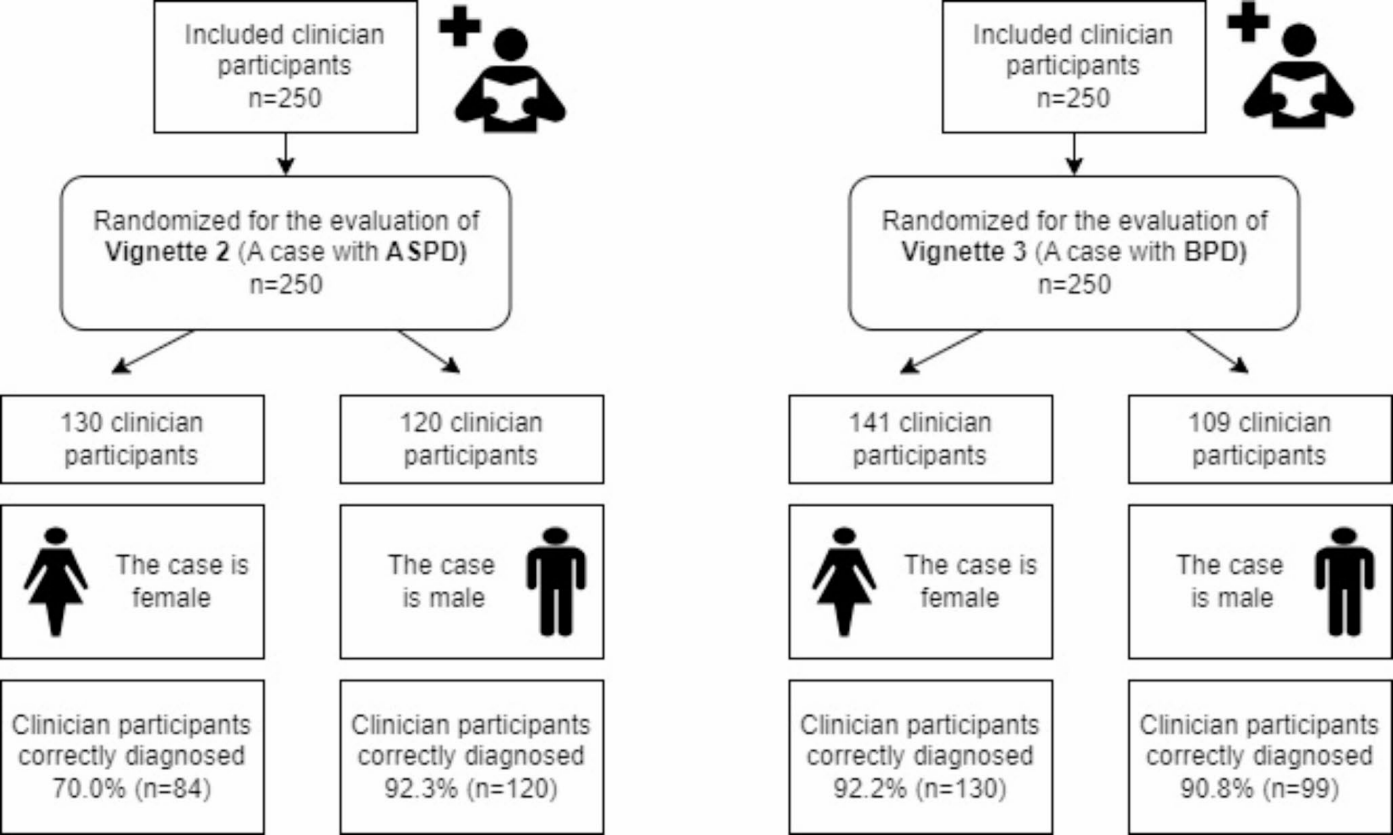
Workflow of the study survey ASPD: Antisocial personality disorder. BPD: Borderline personality disorder

There were 257 participants who volunteered to participate in the study and filled out the forms completely. Seven participants could not correctly diagnose the first vignette, and they were excluded from the analysis. Analyses were completed with data from a total of 250 participants. See Fig. 1 for workflow.

The mean age of the participants was 33.9 ± 6.8 years (minimum 25, maximum 66). 64% were female (*n* = 160), and 36% were male (*n* = 90). 36.4% were psychiatry residents (*n* = 91), and 63.6% were psychiatrists (*n* = 159). The average duration of participants’ professional experience, including residency, was 7.8 ± 6.5 years (minimum 1, maximum 42). Minimal personal information was requested to encourage participation and maintain anonymity. The IP addresses provided by the survey link were checked using the website https://whatismyipaddress.com/; most participants were from 39 cities in Turkiye (96.8%, *n* = 242), while the remaining portion (3.2%, *n* = 8) were from Germany, Azerbaijan, the Netherlands, and Italy. The majority of psychiatrists and psychiatry residents from Turkiye who participated in the research were from Istanbul (35.1%, *n* = 85), Ankara (17.4%, *n* = 42), and Izmir (9.5%, *n* = 23). Characteristics of participants are shown in Table 1.

**Table 1:** Gender-stratified characteristics of participants

Variable	Female (*n* = 160)	Male (*n* = 90)
Age (mean ± SD)	33.7 ± 6.4	34.2 ± 7.5
Psychiatry specialist (n, %)	100 (62.5%)	59 (65.6%)
Psychiatry residents (n, %)	60 (37.5%)	31 (34.4%)
Years of professional experience (mean ± SD)	7.6 ± 6.1	8.2 ± 7.2

### Comparement of randomly assigned groups

There were no differences in age, gender, professional status, and professional experience between participants who viewed the vignettes as female or male. For Vignette 2, there were no differences in age (t = 0.576, *p* = 0.565), years of professional experience (t = 0.238, *p* = 0.812), gender (χ²=0.712, *p* = 0.399), or being a specialist or assistant (χ²=0.195, *p* = 0.658). Similarly, for Vignette 3, there were no differences in age (t = 0.557, *p* = 0.578), years of professional experience (t = 0.802, *p* = 0.423), gender (χ²=0.741, *p* = 0.389), or being a specialist or assistant (χ²=0.007, *p* = 0.932) in the randomly divided groups.

### Evaluation of antisocial personality disorder case

Regarding the male antisocial personality disorder (ASPD) case, 92.3% (*n* = 120) of the subgroup correctly diagnosed it, while 3.6% (*n* = 9) diagnosed it as Borderline Personality Disorder (BPD), and 0.4% (*n* = 1) diagnosed it as Narcissistic Personality Disorder (NPD). For the female antisocial personality disorder (ASPD) case, 70% (*n* = 84) of the subgroup correctly diagnosed it, while 29.2% (*n* = 35) diagnosed it as BPD, and 0.8% (*n* = 1) diagnosed it as Narcissistic Personality Disorder (NPD).

### Evaluation of borderline personality disorder case

**Fig. 2:**
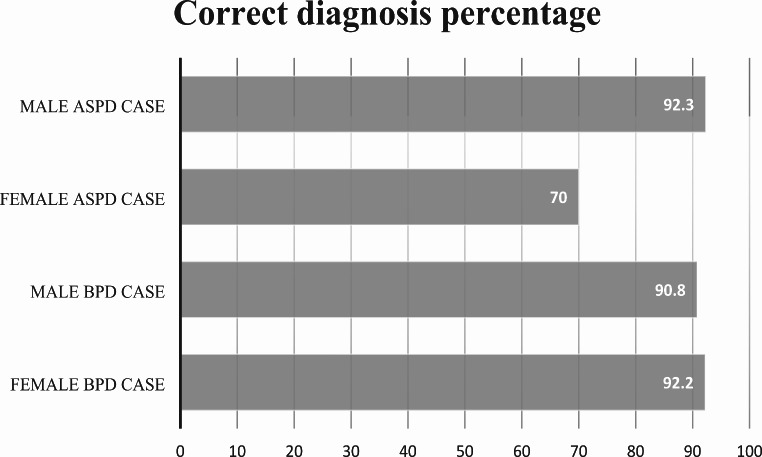
Correct diagnosis percentages of the cases with borderline personality disorder (BPD) and antisocial personality disorder (ASPD)

For the male borderline personality disorder (BPD) case, 90.8% (*n* = 99) of the subgroup correctly diagnosed it, while 9.2% (*n* = 10) thought it was ASPD. For the female BPD case, 92.2% (*n* = 130) of the subgroup correctly diagnosed it, while 3.5% (*n* = 5) thought it was ASPD, 2.8% (*n* = 4) thought it was Histrionic Personality Disorder (HPD), and 1.4% (*n* = 2) thought it was NPD. See Fig. 2 for a summary.

### Evaluation of gender bias

The probability of misdiagnosis for the female ASPD patient was approximately 5.1 times higher compared to the male ASPD patient (Odds ratio = 5.143, 95% CI = 2.419–10.932, z = 4.257, *p* < 0.001). There was no statistically significant difference in the probability of misdiagnosis between female and male patients with BPD (Odds ratio = 1.194, 95% CI = 0.488–2.923, z = 0.388, *p* = 0.698). See Table 2 for an overview.

**Table 2:** Risk analysis of gender bias

Psychiatrists misdiagnosing ASPD	Male patients (*n* = 130)	Female patients (*n* = 120)	Odds ratio (Female patients to male patients)	95% CI	
				Lower	Upper
	7.7%	30.0%	5.143	2.419	10.932
Psychiatrists misdiagnosing BPD	Male patients (*n* = 109)	Female patients (*n* = 141)	Odds ratio (Male patients to female patients)	95% CI	
				Lower	Upper
	9.2%	7.8%	1.194	0.488	2.923

## Discussion

BPD and ASPD are two personality disorders claimed to have a significant sex ratio difference and can be confused with each other because of their similar characteristics. (APA 2022; Amerio et al. 2023; He et al. 2022). The main focus of this study was to examine the impact of gender bias concerning the diagnosis of ASPD and BPD. The results, with statistical significance, demonstrated that a female case with ASPD was 5.1 times more likely to be misdiagnosed than a male case with ASPD, with almost all misdiagnosed females receiving a BPD diagnosis. In contrast, all incorrect diagnoses of male cases with BPD labeled the patients as having ASPD. Thus, the primary issue identified in this study was the tendency for female cases to be misdiagnosed with BPD instead of receiving a proper diagnosis of ASPD, while male cases were more frequently diagnosed with ASPD regardless of diagnostic accuracy. Importantly, there was no statistically significant difference in the probability of misdiagnosis between female and male patients with BPD (Odds ratio = 1.194, 95% CI = 0.488–2.923, z = 0.388, *p* = 0.698). Although unlike our first hypothesis, male BPD cases were not misdiagnosed at a higher rate compared to female BPD cases, the overall pattern suggests a gender disparity in the diagnosis of personality disorders, with more women being diagnosed with BPD and more men being diagnosed with ASPD.

Harder recognition of female ASPD patients by clinicians may occur due to clinicians’ bias (evaluation bias) or bias in diagnostic criteria (criterion bias) (Morey et al. 2002). Particularly when ASPD is concerned, one of the main reasons for such bias seems to stem from the DSM criteria, which uses gender-based trait dimensions to diagnose personality disorders. The effect of gender on symptoms is not an artificial fact but a factor that partially determines certain psychopathological clusters. For example, irritability/aggression and disregard for the safety of others are also more common in the normal population in men than in women(APA 2022). Therefore, although ASPD may present in different ways in men and women, emphasizing aggressive elements, especially conduct disorder, in the definition of ASPD may lead to underdiagnosis of women. Sprock and colleagues (1990) showed in their study that some disorders’ diagnostic criteria could be seen as prototypically masculine or feminine. That can be interpreted as evidence of “gender weightings” within the criteria, like labeling antisocial or sadistic as masculine and dependent or histrionic as feminine (Sprock et al. 1990). In addition, all clinicians who misdiagnosed the male BPD cases had diagnosed the cases with ASPD. In other words, male ASPD cases were not only more easily recognized, but even when male patients did not meet the criteria, they were overdiagnosed with ASPD. Indeed, when ASPD diagnosis is compatible with the gender of the patient with a masculine gender role stereotype association, it seems to increase the diagnosis probability for the clinician, and the presence of gender bias in the diagnosis of ASPD is supported.

Bias against ASPD has two main consequences. First, diagnosing BPD instead of ASPD among females may result in ineffective treatment attempts. Thus, it may raise the labeling of BPD as incurable. As mentioned earlier, people with BPD already face high levels of prejudice and discrimination from the community and medical professionals (Sheppard et al. 2023). Moreover, this may even cause self-stigma by perceiving neutral or ambiguous warnings about themselves and the world as unfavorable in people with BPD (Grambal et al. 2016). As a result, both initiating and maintaining BPD treatment can become challenging. Second, diagnosing ASPD instead of BPD among males may lead the patient to not getting proper treatments. ASPD patients evoke strong emotions that often lead to exclusion from treatment programs. Diagnosed with ASPD is often an exclusion criterion for admission to mental healthcare. Even if patients with ASPD seek help for comorbid disorders, they do not get access to treatment (van Dam et al. 2022).

Both underdiagnosis of gender-role inconsistent diagnoses (female ASPD) and overdiagnosis of gender-role-consistent diagnoses (male ASPD) seem to be associated with gender bias. Henry and Cohen, in a similar study they conducted in 1983, found no gender difference when diagnosing BPD. They argued that the subject response was not a true reflection of perceived gender characteristics, as case studies were fictitious by design (Henry and Cohen 1983). That means the gender difference in our findings might be even higher in actual clinical interviews.

Biased diagnostic constructs, biased diagnostic criteria, and biased diagnostic thresholds, which Widiger defines as gender bias paths in the diagnosis of personality disorders, may also be a part of this problem (Widiger 2011). Biased diagnostic constructs involve categorizing women’s behaviors as pathological, influenced by sexist beliefs or stereotypes. Biased diagnostic criteria imply that behaviors conforming to traditional gender roles may be viewed as less pathological. Diagnosis thresholds may be biased if there are different criteria for diagnosing women compared to men, possibly due to varying assumptions about the level of impairment associated with certain personality traits or behaviors (Skodol and Bender 2003). For this reason, many studies continue on the highly criticized DSM diagnostic criteria. In a study by Samuel and Widiger, it was revealed that employing a dimensional model of personality, such as the Five Factor Model, resulted in a reduction of gender bias when diagnosing personality disorders compared to using the categorical criteria outlined in the DSM (Garb 2021; Samuel and Widiger 2009).

The present study found no relationship between the clinician’s correct diagnosis of cases and the clinician’s gender. Even though there are some evidence supporting women having less sexist attitudes (Yılmaz-Karaman et al. 2023), both female and male psychiatrists may internalize sexism. Besides, diagnostic systems may serve as products of societal gender ideologies. We may interpret that the results stem from both individual and structural attitudes toward gender ideology.

Moreover, even without factors such as clinical judgment and the validity of clinical tools, inequalities due to gender bias in health care may persist. As noted by Cook and colleagues, when and how individuals decide whether to initiate or maintain mental health services, community and family factors such as social networks that influence care, the differential impact of health care policy and funding may already show differences due to gender biases (Cook et al. 2018; Garb 2021). As a result, gender bias regarding personality disorders, which was first mentioned by Kaplan, who claimed that the experts who prepared the DSM-III labeled women as pathological because of certain gender role stereotypes and some masculine-based assumptions about which behaviors are healthy, is unfortunately still a widely debated issue today (Kaplan 1983).

Overall, gender bias exists in the diagnosis of personality disorders, although the extent to which different prevalence rates represent true gender differences remains an unanswered question. Future research should try to determine these disorders’ true prevalence, understand gender bias better, improve diagnostic methods, and reduce other contributing factors. The results of this study encourage clinicians to reflect when diagnosing a personality disorder. A crucial initial step in mitigating biases is ensuring that the diagnostic criteria do not contribute to such issues. Therefore, employing diagnostic criteria such as the Five Factor Model may offer advantages. However, further research is necessary to explore this potential benefit.

Gender bias in clinical diagnoses is a form of sexism. Since clinicians are part of society, and society’s values are internalized by them, too. Turkiye, located between Europe and the Middle East, has been struggling with violence against women and femicide, in addition to the gender gap in professions (Yılmaz-Karaman et al., 2022a; Yılmaz-Karaman et al. 2022b). According to the Global Gender Gap Report 2023, Turkiye ranks 129 out of 146 countries regarding gender equality (World Economic Forum 2022). Turkiye’s patriarchal culture and related gender-role stereotypes may affect the results of the present study. Since being aggressive and hostile is more commonly associated with male gender; the discrepancy in diagnosing female patients with ASPD might be related to internalized gender stereotypes.

### Strengths and limitations

The study employed a robust methodological approach by utilizing randomization, which minimizes potential biases and ensures unbiased allocation of participants. Additionally, using standardized facts from the DSM for case materials enhances the reliability and validity of the study, as it aligns with established diagnostic criteria. Participants were not informed about the study’s focus on gender-based bias to reduce social desirability bias and facilitate more genuine responses.

However, despite these strengths, there have been criticisms regarding the external validity of case vignettes. The limited information in these brief cases may lead clinicians to rely on stereotypes when formulating their responses. Furthermore, the narratives may not capture the complexities observed in actual patients and lack the comprehensive behavioral observations and other relevant details typically gathered during face-to-face assessments. The present study utilized convenience sampling method, and that makes it difficult to generalize the findings. Another limitation is that the study was conducted online rather than face-to-face. Last but not least, we utilized a binary gender structure to understand and analyze differences. However, gender has a fluid nature, and binary approaches can not be enough to understand it.

## Electronic supplementary material

Below is the link to the electronic supplementary material.

Supplementary Material 1
