# Supplementary material for: Gender bias of antisocial and borderline personality disorders among psychiatrists
Source: Arch Womens Ment Health. 2024 Oct 4;28(3):563–71. doi: 10.1007/s00737-024-01519-0 (PMC12092533; doi:10.1007/s00737-024-01519-0)
Supplement: Supplementary file 1 — Supplementary Material 1 [file 737_2024_1519_MOESM1_ESM.docx]

SUPPLEMENT- Vignettes

Vignette 1

Vignette 1 was a 26-year-old male/female, single, living with his/her family. He/she did not have a job. He/she claimed that he/she could control other people's behaviors by taking a breath, could read people's minds, and felt that they have been followed and watched. He/she has been hearing voices telling him/her, he/she would be killed soon because he/she had caused others to commit crimes. These voices usually started after waking up and continued intermittently throughout the day. The patient, who was admitted to a psychiatric clinic with these complaints, showed improvement in symptoms after treatment. However, after discharge from the clinic, he/she struggled to work in a job later, and his/her social relationships became limited. he/she has been spending most of their time at home.

Vignette 2

Vignette 2 was a single 26-year-old male/female who dropped out of high school, living alone with one child. His/her child was taken care of by his/her grandmother. he/she was referred to a psychiatric clinic due to a suicide attempt. The patient had a history of three previous suicide attempts.

The patient’s history revealed that he/she never knew his/her father and he/she was taken care of by his/her mother until the age of 9. After seriously injuring his/her younger sibling by throwing them out of a window, the patient was placed in an orphanage and later at his/her grandmother's house. The patient began experiencing adjustment problems in school, engaging in fights with other children, and torturing animals. he/she started using drugs at the age of 10 and began smoking at 12. he/she did not take care of their siblings at home and did not listen to his/her grandmother. he/she frequently stayed out late at night. he/she and his/her friends shoplifted. When caught by the shop owners, he/she usually avoids punishment through his/her grandmother's intervention. he/she could not finish high school due to absenteeism. he/she worked as a store clerk or sales consultant, but these jobs did not last long. At 23, he/she unexpectedly became a parent, but he/she did not care for the child, and his/her grandmother began caring for them. He/she had a history of high-dose drug use and previous treatments due to depression and suicide attempts. During his/her hospital stays, he/she initially got along well with other patients and followed the facility's rules. Over time, the patient would argue with staff if his/her requests were unmet and had difficulty complying with rules. The patient caused groupings among other patients. The patient was discharged early due to non-compliance with the ward's conditions. During the mental status examination, the patient was appropriately dressed. He/she exhibited a friendly attitude towards the interviewer and made jokes during the examination. His/her attention was adequate, and orientation to person, place, and time was appropriate. No memory deficits were detected. His/her speech was coherent, purposeful, and at an average pace. No perceptual abnormalities were observed.

Vignette 3

Vignette 3 was a 25-year-old female/male, the youngest of four siblings, single, employed, and also, he/she was studying at a university through distance education. He/she was brought to the emergency room by his/her friend due to suicidal thoughts. In the patient's history, it was learned that his/her father had a diagnosis of alcohol use disorder, and his/her parents divorced when he/she was three years old, allegedly due to his/her father's physical abuse towards the patient, his/her siblings, and his/her mother. When the patient was ten years old, he/she was sexually abused by someone five years older than him/her. He/she stated that he/she started using illegal substances during adolescence and, at 17, overdosed on substances due to a problem with his/her partner. For the past few months, the patient has been in a depressed mood, sleeping excessively, gaining weight, experiencing anxiety, and having difficulty concentrating. He/she reports that his/her symptoms worsened when he/she was alone. He/she had long-term fantasies about killing his/her ex-partner, desired self-harm, and cut their body with a razor a few times. He/she states that after two years of dating, he/she called his/her ex-partner and threatened to commit suicide if they did not get back together, and when they got back together, he/she could not control his/her anger and physically attacked his/her ex-partner.
